# Venereal Warts

**Published:** 1909-07-24

**Authors:** 


### DERMATOLOGY.

### VENEREAL WARTS.

Venereal warts, or condylomata acuminata, are lobulated or papillated growths which may appear about the anus and genitals in persons of both sexes, usually in association with gonorrhœal discharges. They may occur, however, in pregnant women who have not been infected with gonorrhœa. They have nothing to do with syphilis, and are to be distinguished from mucous tubercles or syphilitic condylomata, which are always flatter, with no marked tendency to become lobulated, and nearly always associated with other signs of syphilis. It should be remembered, however, that children with congenital syphilis are liable to develop condylomata at the anus when they are about two years of age, and that these specific condylomata may become considerably lobulated. It is almost certain that the so-called venereal warts, or condylomata acuminata are a variety of verruca vulgaris, and it is probable that they arise as the result of a local infection by simple pus organisms. It is claimed by some writers that they are always preceded or accompanied by common warts upon the fingers or elsewhere.

In the female these growths occur chiefly about the anus, on the vulva, and on the contiguous parts of the thighs; the whole of this area may be more or less thickly covered with cauliflower-like excrescences. Often the growths are bathed in a foul-smelling secretion.

In the male the warts are situated in the furrow between the glans and the prepuce, but they may be so luxuriant in growth that the whole preputial sac is filled with vegetations, and they may even spring from the mouth of the urethra.

These growths may sometimes be mistaken for epithelioma, but they are to be distinguished by the absence of any infiltration of the base, and by the fact that there is no ulceration. They are generally very persistent, except in the case of pregnant women, when the growths usually disappear after the birth of the child.

Histologically these growths show a very marked increase in the number of cells in the prickle-cell layers, with abundant nuclear divisions as evidence of rapid multiplication of cells. The interpapillary processes are much hypertrophied, elongated, and branched, and between them are numerous dilated blood-vessels and lymphatics in the sub-epithelial tissues. This greatly increased vascularity accounts for the freedom with which the growths bleed if they are treated surgically or by cauterisation. The treatment of condylomata acuminata consists in the first place of procuring and maintaining absolute cleanliness of the parts. The growths and surrounding structures should be bathed several times a day with a weak solution of tar, or with a boracic acid lotion used hot, followed by powdering freely with a 2-per-cent. salicylic acid starch powder. In some cases continued treatment on these lines will suffice gradually to dry up the growths. But if these means fail it may be necessary to have recourse to surgical measures. The patient should be put under an anæsthetic, and the vegetations snipped off with scissors close to their base. Bleeding will be profuse, but it is readily controlled by pressure. After removal with scissors, the base should be cauterised with the thermo cautery.

